# From Animal Models to Clinical Trials: The Potential of Antimicrobials in Multiple Sclerosis Treatment

**DOI:** 10.3390/biomedicines11113069

**Published:** 2023-11-16

**Authors:** Muhammad Faraz Raghib, Evanthia Bernitsas

**Affiliations:** 1 Department of Neurology, Wayne State University School of Medicine, Detroit, MI 48201, USA; farazraghib@wayne.edu; 2 Sastry Neuroimaging Laboratory, Department of Neurology, Wayne State University School of Medicine, Detroit, MI 48201, USA

**Keywords:** multiple sclerosis, antimicrobials, antibiotics, treatment, autoimmune disease, animal models, Epstein–Barr virus, antivirals, experimental autoimmune encephalomyelitis, microbiota

## Abstract

Multiple sclerosis (MS) is a chronic, autoimmune, demyelinating disease of the central nervous system (CNS). Microbes, including bacteria and certain viruses, particularly Epstein–Barr virus (EBV), have been linked to the pathogenesis of MS. While there is currently no cure for MS, antibiotics and antivirals have been studied as potential treatment options due to their immunomodulatory ability that results in the regulation of the immune process. The current issue addressed in this systematic review is the effect of antimicrobials, including antibiotics, antivirals, and antiparasitic agents in animals and humans. We performed a comprehensive search of PubMed, Google Scholar, and Scopus for articles on antimicrobials in experimental autoimmune encephalomyelitis animal models of MS, as well as in people with MS (pwMS). In animal models, antibiotics tested included beta-lactams, minocycline, rapamycin, macrolides, and doxycycline. Antivirals included acyclovir, valacyclovir, and ganciclovir. Hydroxychloroquine was the only antiparasitic that was tested. In pwMS, we identified a total of 24 studies, 17 of them relevant to antibiotics, 6 to antivirals, and 1 relevant to antiparasitic hydroxychloroquine. While the effect of antimicrobials in animal models was promising, only minocycline and hydroxychloroquine improved outcome measures in pwMS. No favorable effect of the antivirals in humans has been observed yet. The number and size of clinical trials testing antimicrobials have been limited. Large, multicenter, well-designed studies are needed to further evaluate the effect of antimicrobials in MS.

## 1. Introduction

Multiple sclerosis (MS) is a chronic, autoimmune, central nervous system (CNS) disease with both an inflammatory and neurodegenerative component. The disease presents a global concern and has a worldwide distribution, with frequent and dynamic changes as more demographic data from different parts of the world become available [1,2]. It is mainly caused by activated T- and B-cells, with growing evidence of a significant contribution from the cells of the innate immune system, such as dendritic cells, astrocytes, and microglia, induced by environmental factors in genetically predisposed individuals [3,4,5,6]. However, the precise etiology of MS remains unknown, and growing evidence supports multifactorial etiopathogenesis. 

Infections, either bacterial or viral, have long been implicated, but their exact role is still unclear. The effectiveness of interferon therapy and the elevated detection of viral antibodies and genetic material in the MS population compared to healthy individuals suggest a possible viral contribution [7]. Certain viruses, such as the human herpes virus, and especially Epstein–Barr virus (EBV), have been suggested to play an important role in the causative pathway leading to MS development, either directly or via autoimmune mechanisms [8]. Being symptomatic with infectious mononucleosis or testing positive for antibodies against nuclear EBV antigens increases by approximately 30-fold the risk of being diagnosed with MS, a finding that is backed by the fact that being EBV-negative can be protective [9]. EBV-induced B-cell immortalization is believed to be a key factor leading to MS [10,11]. Despite evidence of an infectious contribution, the interplay between infections, immune pathways, and MS is unclear and still developing. Multiple mechanisms, such as molecular mimicry between the myelin antigen and the pathogen and the entrance of the pathogen into the CNS via an impaired blood–brain barrier with subsequent neuroinflammation, have been hypothesized.

MS is an immune-mediated and not an infectious disease. Therefore, any role that antimicrobials could have as an MS treatment is via their effects on the immune system. While there is currently no cure, antimicrobials have been studied as potential treatment options due to their ability to regulate the immune process. Their immunomodulatory role, either in combination with disease-modifying therapies (DMT) or as a monotherapy, is still being explored and is a topic of ongoing research. 

The mechanism of action by which antimicrobials might affect the course of MS is complex. Over the last four decades, research studies have demonstrated that antimicrobials target several different pathways. Their anti-inflammatory and antioxidant effects might contribute to favorable disease outcomes, such as annualized relapse ratio (ARR), disability progression measured by the expanded disability status scale (EDSS), and MRI metrics, including the number of gadolinium-enhancing lesions and the number of new or enlarging lesions [12,13]. Moreover, the functional alteration of the gut microbiome by certain antimicrobials, a key player in the development of several autoimmune diseases, represents another mechanism by which antimicrobials might be useful in the management of MS. Rifaximin, a poorly absorbed antibiotic, can induce eubiotic changes in the intestinal ecosystem, promoting a positive modulating effect on the gut microbiota and leading to a favorable outcome [14,15,16,17]. Given its immuno-regulatory functions, such as regulation of the blood–brain permeability, microglia activation, limitation of astrocyte pathogenicity, and expression of myelin genes, the microbiota was found to play a central role in the pathogenesis of experimental autoimmune encephalomyelitis (EAE) [18,19,20,21,22].

In the last two decades, we observed an explosion of the FDA-approved DMT, providing excellent treatment options for pwMS. However, the treatment response differs greatly between pwMS, and the risk of side effects is still a concern [23,24,25,26]. More robust and well-optimized treatment strategies are required, either via the advent of new therapeutic agents or a combination of existing ones. We aimed to perform a systemic search on the effect of antimicrobials on the disease metrics to improve our understanding of whether and how they can contribute to improved MS outcomes. In this systematic review, we summarize experimental studies in animal models and clinical trials in pwMS, and we discuss the mechanism of action of certain antimicrobials and their potential role in the management of MS. This systematic review aims to critically appraise the currently available literature on the role of antimicrobials in MS outcomes.

## 2. Methods

**Figure 1:**
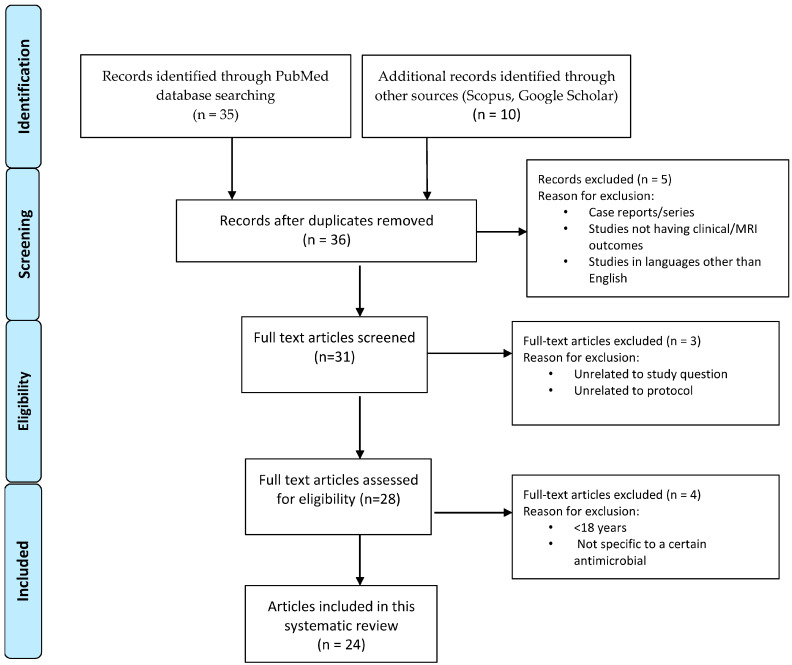
Flow diagram of identification process for eligible studies per PRISMA guidelines.

Following the Preferred Reporting Items for Systematic Reviews and Meta-Analyses (PRISMA) guidelines, two reviewers (MR and EB) independently performed a rigorous and methodical literature search using three different electronic databases (PubMed, Scopus, and Google Scholar) [27,28]. Discrepancies were resolved via discussion until a consensus was reached between the reviewers. The nature of this review did not require Institutional Review Board approval. We adhered to the guidelines of the NHS Centre for Reviews and Dissemination [29]. We used the following word combinations: “antimicrobials”, “antibiotics”, “antivirals”, “animal models”, “experimental autoimmune encephalomyelitis”, “multiple sclerosis”, “microbiome”, “immunomodulation”, “penicillins”, “cephalosporins”, “minocycline”, “tetracyclines”, “doxycycline”, “erythromycin”, “rapamycin”, “azithromycin”, “hydroxychloroquine”, “Epstein Barr virus”, “herpes virus”, “acyclovir”, “valacyclovir”, and “HIV”. In addition, ClinicalTrials.gov, a database from the National Institute of Health, was extensively searched to identify relevant registered and ongoing trials. The search was not restricted to date. Only articles in the English language published in peer-reviewed journals were included, and duplicates were excluded. We excluded case reports, case series, commentaries, and blog articles. All clinical trial designs were included, given the limited number of studies conducted. Articles were disqualified if they did not directly address the study question. To ensure the quality of this review, specific inclusion and exclusion criteria were used. Inclusion criteria included a confirmed diagnosis of CIS or MS per 2005, 2010, and 2017 McDonald criteria, age > 18 years old, antimicrobial use, and clinical or imaging outcomes [30,31,32]. Exclusion criteria included age < 18 years old, studies not specific to a certain antimicrobial, lack of imaging, or clinical outcomes. After the identification of eligible studies, the following data were obtained from each article: author(s)’s name, title, year of the study, sample size, study design, MS type, antimicrobial studied, treatment duration, study outcomes and measures, and length of follow-up. This process reduced the risk of bias and ensured our results would stand against scrutiny. The authors collaborated to present a comprehensive review of the therapeutic effect of antimicrobials and their current and future prospects. Figure 1 depicts the flow of information via the different stages of the identification process for eligible studies.

## 3. Results

### 3.1. Beta-Lactam Antibiotics

Beta-lactam antibiotics are widely used in the management and treatment of bacterial infections. This class includes penicillin (PCN), cephalosporins, carbapenems, monobactams, and beta-lactamase inhibitors. They bind to penicillin-binding proteins, which, in turn, interrupt the terminal transpeptidation process and cause loss of viability and lysis of the bacterial cell. In addition to their antimicrobial effects, beta-lactams have also been shown to modulate the immune response by several mechanisms [33]. One of the mechanisms involves upregulation of the neuroprotective protein, the presynaptic glutamate transporter 1 (GLT1), which removes glutamate from the synaptic cleft, thereby reducing its concentration in the synaptic cleft and thus decreasing the neurotoxic effects of glutamate [34]. It is well-established that excessive activation of the glutamatergic pathway plays an important role in the pathophysiology of MS; therefore, by enhancing the GLT1 expression, beta-lactam antibiotics might offer some degree of neuroprotection. Experiments in rodents and in human T-cells, both in vivo and in vitro, demonstrated that beta-lactam antibiotics modulate T-cell behavior, alter the T-cell gene expression and either upregulate or downregulate the pro-inflammatory T-cell phenotype via covalent binding to albumin [35]. 

Ceftriaxone (CEF), a beta-lactam drug, has been extensively studied as a neuroprotective agent against several glutamate-associated neurologic diseases due to its large volume of distribution [36]. In animal models of Parkinson’s and Alzheimer’s disease, CEF-treated animals showed improvement in memory impairment, downregulation of the tau protein, restoration of cognitive function, and neuronal density [37,38,39]. In a murine myelin–oligodendrocyte glycoprotein-induced (MOG) EAE model, CEF reduces the disease severity by causing a reduction in T-cell activation via the modulation of cellular antigen-presentation and impairment of antigen-specific T-cell migration into the CNS [12,39]. Despite these observations, CEF has not been tested in pwMS yet. 

PCN, another beta-lactam antibiotic, has been studied in two trials in pwMS. A large UK-based case–control study reported that PCN administration of more than 2 weeks within 3 years prior to the appearance of the first symptom of MS was associated with a 50% reduced risk of developing MS [40]. On the contrary, a Danish case–control study of 3259 pwMS reported a higher risk of MS across a wide range of different antibiotics, including PCN, even after a short 7-day treatment course [41].

Interestingly, a more recent case–control study demonstrated allergy as a protective factor for MS. Patients with respiratory tract allergies were more likely to use antibiotics, including PCN, and those with other non-respiratory tract allergies also had a high likelihood of PCN use. Although no direct link was confirmed between PCN use and the risk of MS, PCN may mediate the relationship between allergies and MS. These results suggest that antibiotic use might not be a suitable indicator of bacterial infection to investigate the cause of MS [42]. These results were confirmed by a recent study that demonstrated no relationship between antibiotic exposure and the risk of MS [43].

### 3.2. Tetracyclines

#### 3.2.1. Minocycline (Minocin, Dynacin, Ximino, and Solodyn)

Minocycline is a second-generation tetracycline that can cross the blood–brain barrier at clinically effective levels due to its lipophilic nature. Research on minocycline spans several decades, making it the most extensively studied antimicrobial. Minocycline showed a neuro-protective effect in several neurodegenerative diseases, such as Parkinson’s disease, Huntington’s disease, amyotrophic lateral sclerosis, stroke, and MS [44,45,46]. The signaling mechanisms by which minocycline acts as a neuroprotectant in neurodegenerative diseases are complex, multiple, and mainly involve an anti-inflammatory, antiapoptotic, and anti-oxidative effect [47,48,49,50,51,52]. 

While the exact mechanisms of demyelination and axonal loss are not yet fully known, the literature supports that apoptosis in CNS neurons plays an important role in the pathogenesis of MS [53]. Minocycline is known to upregulate the expression of B-cell lymphoma 2, an anti-apoptotic protein, downregulate the expression of apoptosis-inducing factor, a pro-apoptotic protein, and stabilize the mitochondrial membrane to prevent the release of mitochondrial products from leaking out, thus preventing apoptosis [54,55]. 

The anti-inflammatory role of minocycline in MS is brought by the inhibition of T-cell migration and T-cell release of matrix metalloproteinases (MMPs) that damage the blood–brain barrier [56,57,58]. In a study where levels of different neurotrophic factors produced by peripheral blood mononuclear cells were measured, the levels of nerve growth factor (NGF) were significantly higher in patients who have fully recovered after an MS relapse [59]. Other observations showed that levels of brain-derived neurotrophic factor (BDNF) are lower in pwMS compared to healthy controls and increased post-relapse during the recovery period [60]. Using the EAE mouse model and performing experiments both in vivo and in vitro, Chen and colleagues demonstrated the upregulation of NGF and BDNF by minocycline [61].

These effects of minocycline have been evaluated across a wide range of animal models [62,63,64,65,66]. In the MOG-immunized rats, treatment with minocycline was found to reduce the T cell infiltration into the CNS and block the MMP-2 expression. Furthermore, minocycline-treated rats showed a delayed onset of the disease along with a dramatic reduction in disease activity and severity [62]. 

Minocycline administration in the EAE mouse model, after the disease onset, reduces mean and cumulative EAE scores by reducing T-cell infiltration, both CD4 and CD8, into the spinal cord without changing the cytokine profile [63]. Furthermore, it inhibited MMP activity, decreased MMP-9 production, and reduced the transmigration of T cells across a fibronectin matrix barrier [64]. In addition, minocycline was found to be efficacious in both mild and severe EAE in mice. 

Early MS-related memory impairment is attributed to dental gyrus injury secondary to microglial activation [65]. In a mouse model of EAE, minocycline prevented dentate gyrus injury by inhibiting microglial activation [66]. In the same model, suboptimal doses of minocycline and hydroxychloroquine individually delayed the onset of clinical signs. Impressively, their combination markedly attenuated EAE severity until treatment was stopped. These results were expanded in the Biozzi ABH mice, a model of progressive MS, where the combination of minocycline and hydroxychloroquine successfully altered the chronic phase, which could potentially be beneficial and applicable in the progressive phases of MS in humans [67]. In a spectrometry-based proteomics analysis of an animal model of EAE, half of the minocycline-treated EAE animals exhibited no neurological symptoms on day 14, while the other half had neurological symptoms at the same time point [68]. In the rat model of MOGA-induced EAE, minocycline delayed disease onset, improved electrophysiological conduction of the optic system, rescued retinal ganglion cells (RGC), activated the anti-apoptotic pathways, decreased intraretinal glutamate levels, and decreased the severity of optic neuritis. Interestingly, after surgical transection of the optic nerve, minocycline increased RGC survival [69].

Based on the observations on the animal models of MS, clinical trials have been conducted to further explore the effect of minocycline in pwMS. We identified a total of seven clinical trials. Two studies investigated the effect of minocycline as an add-on to platform therapies, and both produced non-significant results. The first study was a phase II, double-blind, placebo-controlled 9-month clinical trial in which minocycline at a dose of 100 mg twice daily has been proposed as an add-on therapy to improve the efficacy of glatiramer acetate (GA) in relapsing-remitting MS (RRMS) [70]. In this study, the combination of GA and minocycline reduced the total number of T1 gadolinium-enhanced lesions by 63%, the total number of new and enlarging T2 lesions by 65%, the total T2 disease burden and the relapse risk when compared with GA alone. Although these results were non-statistically significant, there was a consistent trend favoring combination treatment over monotherapy. In a more recent study investigating minocycline as an add-on therapy to subcutaneous interferon βeta-1a, minocycline showed a non-significant beneficial effect on the time to first relapse, ARR, and MRI metrics, although there were numerical trends in favor of the combination therapy versus monotherapy with interferon βeta-1a [71].

The first trial evaluating the effect of minocycline on gadolinium-enhancing lesions occurred in 2004. After a 3-month run-in period, 10 subjects with RRMS underwent monthly MRI scans up to month 6. The main outcome was the change in the mean number of gadolinium-enhancing lesions per scan during the 6-month treatment period compared with the run-in period. The study demonstrated significance in the main outcome [72]. Three years later, the same study group reported clinical data on the same number of participants over a 6-month treatment period. Minocycline was found to decrease the ARR, the mean number of gadolinium-enhancing lesions, and the level of inflammatory cytokines during the study duration [13]. In an extension study of 36 months, the investigators assessed the safety and tolerability of minocycline and found it to be safe and well-tolerated. Moreover, additional MRI metrics, such as the mean T2 lesion volume, remained stable, and the percentage of brain volume loss was reduced during the third year of minocycline treatment [73]. 

Two more studies targeted people with clinically isolated syndrome (CIS); the first one explored the risk of conversion to MS, while the second one investigated serum biomarkers. The first was a multicenter, randomized, placebo-controlled clinical trial of 142 participants followed over 24 months (MinoCIS trial). The primary outcome was conversion to MS within 6 months after randomization. Secondary outcomes included conversion to MS within 24 months after randomization and MRI changes at 6 and 24 months. A statistically significant lower risk of conversion from CIS to clinically definite MS was found in the minocycline group compared to placebo over a 6-month period but not over 24 months. The lower risk of conversion remained significant after adjusting for baseline gadolinium-enhancing lesions and spinal cord symptomatology at the onset of the disease [74].

In the second study, the investigators used stored samples from the previous MinoCIS trial and measured serum biomarkers in the same population. Minocycline paradoxically increased the serum neurofilament (Nfl) at month 3, while there was a decreasing trend thereafter toward M6, possibly due to a delayed response. Otherwise, the levels of glial fibrillary acidic protein (GFAP) at M6 and MMP-7 at M1 had significantly decreased compared with the control group, as expected [75]. Another trial has just been completed, and the results have not been published yet. This trial aims to confirm the benefits of minocycline in MS patients who have had a first demyelinating event in the past 180 days and at least possess two brain T2 lesions of a minimum of 3 mm in diameter (NCT04291456).

#### 3.2.2. Doxycycline (Vibramycin D and Periostat)

Doxycycline is a tetracycline antibiotic that is lipid-soluble, so it highly penetrates the blood–brain barrier and acts directly on the CNS. It is rapidly and completely absorbed and is a strong MMP inhibitor [76]. While it has not been tested in animal models, it was evaluated in two clinical trials as an add-on therapy to interferon beta-1a. The first open-label trial of 7-month duration evaluated the efficacy and safety of a combination therapy consisting of intramuscular interferon βeta-1a and oral doxycycline in 15 RRMS participants with breakthrough disease. PwMS during the treatment period showed a reduction in the gadolinium-enhancing lesions, disability, and relapse ratio compared with the pre-treatment period. The combination was safe and well tolerated [77]. On the other hand, in a 6-month double-blinded trial of 60 pwMS, including both RRMS and active secondary progressive MS, no favorable MRI outcomes were observed when doxycycline was used as an add-on therapy to either subcutaneous or IM interferon β-1a. However, this combination was found effective in reducing relapses and improving EDSS scores [78].

Interestingly, in an experimental model of peripheral autoimmune neuritis, doxycycline effectively reduced peripheral inflammation to improve the outcome of this demyelinating disease of the peripheral nervous system, suggesting that doxycycline may be considered a potential pharmacological agent for the management of neuropathies [79]. 

### 3.3. Rapamycin (Sirolimus)

Rapamycin, a macrolide antibiotic produced by Streptomyces hygroscopus, is an immunosuppressant and anti-cytoproliferative drug mainly used to prevent allograft rejection in organ transplantations [80]. The therapeutic effect of the drug in the CNS is due to its ability to cross the blood–brain barrier and exerts an anti-inflammatory effect [81]. In addition to its immunosuppressive effect, it inhibits apoptosis and activates intracellular autophagy via inhibition of the assembly of the subunits of the mammalian target of rapamycin (mTOR) [82,83,84]. The first step in the inhibitory molecular pathway includes inhibition of the mTOR kinase, which regulates the cell cycle; thus, rapamycin halts the antigen-mediated B- and T-cell proliferation [83,84,85]. In the relapsing- remitting EAE mouse model, administration of rapamycin at the disease peak or at the end of the first clinical attack has been shown to ameliorate the clinical course along with reduced demyelination and axonal loss via selective suppression of effector T cell function and expansion of T regulatory cells [85]. Similarly, in the chronic EAE mouse model, administration of the rapamycin to the already ill animals at the peak of their disease (therapeutic approach) ameliorated both clinical and histological manifestations of the disease, while early administration before the appearance of the symptoms (prophylactic approach) prevented the development of EAE. Moreover, it reduces the hyperalgesia by increasing the pain threshold, so it might have an additional benefit in the management of painful dysesthesias in pwMS [86]. Furthermore, in the same animal model, rapamycin was tested as an add-on treatment and was found to increase the immunomodulatory properties of the bone marrow-derived mesenchymal stem cells [87]. Another interesting phenomenon observed in the EAE animal model was the recovery of the gut microbiota to an almost normal level when the mice were treated with rapamycin in combination with MCC950. This observation suggests another mechanism of action for rapamycin, potentially beneficial in preventing and treating MS [88]. 

In pwMS, rapamycin was tested only in one pilot study, while its synthetic derivative, temsirolimus, was tested in a multicenter trial. In a single institution, single-arm study, 8 RRMS patients received a daily dose of rapamycin 2 mg over the 6-month study duration. After obtaining MRI scans and disability outcomes, participants showed a significant reduction in mean plaque area size and maximum area volume, while the minimum area volume decreased but not significantly. Half of the participants showed a non-significant decrease in the EDSS post-treatment, while for the other half, the EDSS remained unchanged. Moreover, genes encoding the T-regulatory cells were upregulated [89]. Temsirolimus, a synthetic derivative of rapamycin that is better absorbed than rapamycin, was tested in phase II, placebo-controlled study in 296 patients with RRMS and active secondary progressive MS. Participants were randomized to three different doses, and the highest dose of 8 mg daily showed a significant decrease of 51% in the number of relapses compared with the placebo group and a significant reduction in gadolinium-enhancing lesions. Despite the above results, a phase III study was not pursued due to the critical risk-to-benefit ratio, given the toxicity observed in the treatment group, consisting mainly of drug-induced hyperlipidemia, stomatitis, menstrual dysfunction, and rash [90,91]. 

### 3.4. Antivirals

Several viruses have been implicated in the pathogenesis and progression of MS, including EBV, cytomegalovirus (CMV), and varicella zoster virus (VZV) [92,93]. Among them, EBV, a herpesvirus that infects more than 90% of the global population, has been extensively studied over the last four decades, and its presence as a potential causative factor for the development of MS has been recently demonstrated [94,95]. The role of EBV in the early pathogenesis of MS is supported by several prospective studies demonstrating that elevated titers of EBV antibodies and EBV infection significantly increase the risk of developing MS later in life. Moreover, pwMS have greater than 99% EBV seropositivity as compared to non-MS patients, while the risk of MS is very low in patients who are EBV- seronegative [96,97,98,99,100]. The mechanism of EBV infection leading to MS onset is still debated. One possible mechanism is that EBV infects B-cells and causes these cells to grow, produce CNS antigen-specific antibodies, and enter the CNS to activate the T cells [101]. These T cells may also be activated by the small heat shock protein alpha B-crystallin induced by EBV in B cells and then recognize the alpha B-crystallin in glial cells in MS lesions [102]. Another possible mechanism is molecular mimicry: the cross-reaction of EBV antibodies with CNS-specific antigens, such as the myelin basic protein, to trigger the onset of the disease [103]. 

Lanz and colleagues reported that a monoclonal antibody produced from a B-cell clone derived from the CSF of an MS patient cross-reacted with the EBV nuclear antigen (EBNA-1) and GlialCAM, a protein found in CNS astrocytes and oligodendrocytes [104]. Further, these antibodies were also found in 20% of pwMS. Despite these findings, the main question is whether these antibodies are merely biomarkers or if they truly have a role in the pathogenesis of MS. 

The B-cell depleting DMT mostly indiscriminately depletes or inhibits all B-cells; since these B cells would also include a subset of EBV-infected cells, these drugs may help lower the EBV load and hence the potential for EBV-induced immunopathological response in MS. For example, ocrelizumab treatment has been shown to reduce the EBV-specific immune response, indicating that the benefit of this drug in MS may be achieved via the removal of EBV antigenic stimulus [105].

Currently, no antiviral drugs are FDA-approved to treat EBV infection in vivo. There is anecdotal evidence that some antiretroviral agents that are also potent inhibitors of EBV lytic infection may suppress MS activity [106]. Interestingly, a comparative cohort study using administrative health databases with a cohort of 21,207 HIV-positive patients and 5,298,496 controls demonstrated a lower risk of developing MS in HIV-positive people compared with the control group, either due to the HIV-induced immunosuppression or to the antiretroviral medications use [107]. These findings led to the conduction of a phase 2a clinical trial investigating the effect of raltegravir (400 mg BID), an HIV integrase inhibitor, on 20 pwMS. The study duration was 6 months and consisted of a 3-month baseline phase followed by a 3-month treatment with raltegravir 400 mg twice a day. The primary outcome was the development of gadolinium-enhancing lesions in the treatment phase compared with the baseline phase, and secondary outcomes included quality of life, EBV shedding, EBV antigens, as well as immunological and inflammatory markers. After completion of the study, no change in the primary or secondary outcomes was reported [108]. Currently, an ongoing study investigates the effect of tenofovir alafenamide, another antiretroviral agent, FDA-approved for chronic hepatitis B virus infection. The study explores its effect in combination with ocrelizumab or rituximab on MS-related fatigue, serum neurofilament light chains, disability, annualized relapse rate, EBV viral load, and titers (NCT04880577). 

Several anti-herpetic drugs that inhibit EBV replication in vitro have been evaluated in EBV-related diseases. These antiviral drugs, including acyclovir, valacyclovir, and ganciclovir, inhibit EBV replication, but they lack efficacy over viral latent infection, which is the main factor in the MS pathogenesis. We identified only one study of ganciclovir on the EAE mouse model. The investigators demonstrated that ganciclovir inhibits the proliferation of microglia and attenuates neuroinflammation. When given before the onset of the disease, it prevented the infiltration of T lymphocytes into the CNS and drastically reduced disease incidence and severity [109]. In pwMS, there are only a few published randomized and placebo-controlled clinical trials of anti-herpetic treatment with non-significant results. Most of these trials were conducted more than two decades years ago. One randomized, double-blinded, placebo-controlled trial investigated the role of acyclovir (800 mg TID) in 60 patients with RRMS [110]. Although acyclovir-treated patients showed 34% fewer exacerbations compared with placebo during the study duration, this difference has not reached statistical significance. However, in subsequent subgroup data analysis where patients were grouped according to exacerbation frequencies, acyclovir-treated patients had a reduced mean annualized relapse rate of 0.44, while the corresponding placebo group showed an increased annual exacerbation rate of 0.37 during the study (*p* = 0.024). 

Two clinical trials investigated valacyclovir in pwMS. In a phase 2, placebo-controlled, double-blind, randomized study, Bech and colleagues investigated the effect of valacyclovir on the MRI-evident lesions in 70 RRMS with two or more relapses within 2 years before enrollment. Although valacyclovir treatment did not reduce the formation of new lesions, it had a significant effect on a subgroup of patients with high levels of disease activity. Valacyclovir-treated patients with more than one active lesion at baseline had 2.0 lesions per scan compared with 6.5 lesions per scan in the placebo group (*p* = 0.025), and the proportion of scans with no activity was 28% in the valacyclovir and 5% in the placebo group (*p* < 0.001). This study has mainly focused on MRI metrics and has not investigated any clinical outcome [111]. The second study was a double-blind, placebo-controlled trial of a 2-year duration. Fifty-eight pwMS were randomized to receive valacyclovir (3000 mg daily) or placebo for the study duration. The primary outcome was disease progression, and secondary outcomes included time to first attack, time to withdrawal, and attack rate. No significance was achieved, but some positive trends were noted in both primary and secondary outcomes. Similarly, no significant effect of the drug was demonstrated on MRI measures. Its safety profile was excellent, and no discontinuations were observed due to drug toxicity [112]. Furthermore, an ongoing study of 36-week duration, designed for pwMS on natalizumab, investigates the effect of famciclovir as an add-on therapy on EBV shedding in saliva, EBV serological markers, and EBV viral replication in blood (NCT05283551).

### 3.5. Hydroxychloroquine (HCQ)

In preclinical trials, this antimalarial drug was tested in the EAE animal model and was shown to reduce microglial activation and attenuate the severity of the disease [113]. Using in vitro cell culture assays, the mouse lysolecithin spinal cord model, and a combination of HCQ and indapamide, Brown et al. reported an attenuation in axonal injury, decrease in lipid peroxidation, and inhibition of microglia activity, therefore mitigating the substrates of progression in MS [114]. In the mouse model of acute and chronic EAE, a combination of suboptimal doses of HCQ and minocycline suppressed clinical manifestations of EAE until treatment was stopped [67].

We found only one clinical trial in testing HCQ in pwMS. This is a single-arm, 18- month, phase II futility trial in 35 pwMS of primary progressive type (PPMS). The primary endpoint of this study was the 25-foot timed walk (T25FW), a performance test of lower extremity mobility, which was measured between 6 and 18 months of follow-up. Given that only 8 of the 35 participants experienced worsening, the investigators concluded that treatment with HCQ resulted in reduced disability worsening in PPMS [115].

### 3.6. Other Antimicrobial Agents

Chlamydia pneumoniae is known to be present in the CSF in a subset of pwMS and has been hypothesized to even serve as a contributing factor in the disease pathogenesis [116]. Macrolides are antibiotics commonly used in the management of chlamydial infections. Despite their anti-inflammatory effects, animal studies are scarce with discouraging results. In the EAE mouse model, clarithromycin and azithromycin similarly inhibited nitric oxide (NO) production, NO synthase mRNA, and protein expression in vivo and in vitro and subsequently aggravated EAE [117]. 

In pwMS, the effect of azithromycin as a combination therapy with rifampin was tested in a single double-blinded, placebo-controlled trial of RRMS patients who showed Chlamydia pneumonia gene in their CSF with at least one gadolinium-enhancing brain lesion on baseline MRI scan. Participants were randomized to azithromycin and rifampin group or placebo. The primary outcome measure, the number of enhancing lesions, was not significantly affected by the combination therapy. However, in a post hoc analysis, three of four participants who received antibiotics showed a decrease in the volume of enhancing lesions. Moreover, the antibiotic group had significantly less parenchymal volume fraction loss compared to the placebo group [118]. 

In another study, 28 pwMS were recruited and were treated with three cycles of oral clarithromycin, 6 weeks each. Following the conclusion of the study, no significant difference was found in the EDSS and relapse rate between the treatment group and placebo [119].

The most recent development in the continuously evolving landscape of antimicrobials and MS is a placebo-controlled, currently enrolling trial exploring the effect of vancomycin on the gut microbiota composition, peripheral immune function, and MRI metrics (NCT05539729). The results of this trial will be available in 2025 and may improve our understanding of the interplay between antibiotics and MS.

Overall, several antimicrobials have been tested in animal models, resulting in an improved understanding of their complex mechanism of action. Clinical trials for some of the antimicrobials that have been completed showed excellent safety profile and good tolerability, while other clinical trials are still ongoing. 

**Figure 2:**
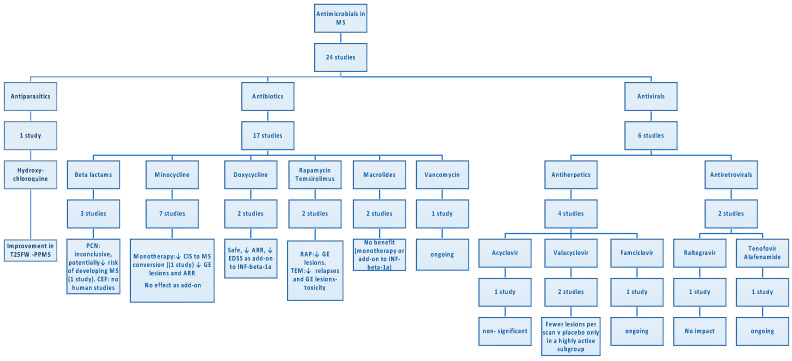
Snapshot of antimicrobials in pwMS based on clinical studies. ARR = annualized relapse ratio, CEF = ceftriaxone, CIS = Clinically isolated syndrome, EDSS = expanded disability status scale, GE = gadolinium-enhancing, INF-beta-1a = interferon beta 1 alpha, MS = multiple sclerosis, PCN = penicillin, PPMS = primary progressive multiple sclerosis, pwMS = people with multiple sclerosis, RAP = rapamycin, TEM = temsirolimus, T25FW = timed 25-foot walk.

Table 1 summarizes the mechanism of action and the effect of antimicrobials on the animal models and study participants. Figure 2 maps out the number of clinical trials for each category of antimicrobials and the main findings of each study.

The included studies underwent a rigorous risk of bias assessment by the authors, according to the Cochrane Handbook for Systematic Reviews of Interventions version 5.1.0 [120]. Table 2 summarizes the risk of bias assessment for each included study. The assessment categories include random sequence generation, allocation concealment, blinding of participants and personnel, blinding of outcome assessment, incomplete outcome data, selective reporting, and other biases. Across most of the included studies, there was a low risk of bias associated with the domain of incomplete outcome data, whereas blinding of participants and personnel emerged as a common challenge, with eleven out of twenty-one studies demonstrating a high risk of bias in this domain. Ongoing clinical trials were not included in the assessment due to a lack of published data. 

## 4. Discussion

Over the last decade, our understanding of MS has greatly advanced as new discoveries, including biomarkers, metabolic pathways, and genetic influences, were implicated in the etiology and pathobiology of MS. Various environmental factors became key players, necessitating further research to determine their potential influence on MS risk, severity, and progression of the disease, as well as treatment approaches [121,122,123,124,125]. 

In recent years, the explosion of DMT targeting disease activity and progression has offered new hope for achieving better outcomes. However, the available FDA-approved DMT, including immunomodulatory and immunosuppressive drugs, are not curative. Despite their efficacy in the early stages of RMS, their efficacy is minimal, if any, at later stages or in progressive MS, where neurodegeneration becomes the dominant factor. Currently, there are no therapeutic modalities to reverse demyelination or neurodegeneration, even to a modest degree. Their safety profile might be concerning, and the possibility of serious side effects may lead to non-compliance or even treatment discontinuation [126,127,128,129]. Subsequently, there is an unmet need to improve outcomes in MS, especially in the progressive forms. Antimicrobials have been explored to offer an alternative, non-expensive pathway in the management of MS.

In this systematic review, we identified 24 clinical trials with different designs and cohorts examining the effect of anti-infective agents in pwMS. Of note, only a very small number of antimicrobials was tested in both animals and humans. Starting with the beta-lactams, this drug group might potentially serve as neuroprotectants by decreasing glutamate-based excitotoxicity via the increased expression of glutamate transporters. It is still controversial whether PCN confers a reduced risk of developing MS. However, its effect on clinical and MRI outcomes has never been investigated in pwMS. Another drug of the group, CEF, showed promising results in animal models, but it has never been tested in clinical trials. Based on the literature to date, macrolides (azithromycin, clarithromycin) yielded discouraging results in both animal models and humans, making them an unlikely target for further study. However, it should be noted that azithromycin was tested as an add-on therapy to another antibiotic, but the study was limited by the small sample with only four patients in the antibiotic group. In a small study of only 6-month duration and without a control group, rapamycin was found to improve MRI outcomes and being well tolerated, while its synthetic derivative, temsirolimus, was found to be highly efficacious and highly toxic as well, limiting further investigation and subsequent use. 

Minocycline has been the most extensively and most promising antibiotic investigated in pwMS. Several studies to assess the effects and safety of minocycline as a monotherapy in pwMS have already been performed and pointed to its therapeutic effect in MS [130]. Despite the encouraging results from the clinical trials, their small sample size, the lack of a control group, and the fact that all were single-institution trials with the same patient population warrant careful interpretation of the results. Moreover, minocycline was tested in a large multicenter trial with a follow-up duration of 24 months, and it showed a significant reduction in conversion to clinically definite MS. The relatively small size, the inability to enroll the total planned number of 154 participants, and the censoring of the data from 22.5% of participants might have limited the power of this study at 24 months. Based on this study, minocycline might be of some use in CIS, and its effect should be further validated in larger, well-designed clinical trials. However, taking into consideration the 2017 updated McDonald criteria, a subgroup of people who were diagnosed with CIS may be qualified for an MS diagnosis at present. Subsequently, no definite conclusions on the effect of minocycline in people with CIS can be made. Paradoxically, minocycline as an add-on therapy to either GA or interferon beta-1a yields discouraging results. On the contrary, doxycycline as an add-on therapy to interferon beta-1a decreased disability scores and relapse ratio, while the effect of this combination on the MRI metrics is still controversial. Despite the small sample and the short treatment duration, the results provided preliminary evidence of the potential beneficial effect of this combination. Another class of antibiotics, the macrolides, showed no benefit either in the short or long term, making a causative connection between Chlamydia and MS very unlikely.

Interestingly, HCQ, the only antiparasitic drug tested, had a positive effect on T25FW in PPMS, even though this study was limited by the small number of participants and the lack of additional endpoints or disability outcomes. Furthermore, HCQ was shown to decrease the serum neurofilament light chain and the glial fibrillary acidic protein in pwMS [131]. It is noteworthy that a combination of HCQ and minocycline in suboptimal doses in the chronic EAE mouse model ameliorated the disease, suggesting a possible new treatment in progressive MS and prompting investigators to test this combination treatment, possibly in higher doses in pwMS. Regarding their safety profile, except for temsirolimus, antimicrobials were well tolerated, and their reported adverse events were mild and transient.

Despite the encouraging results of antivirals in animals, these results have not been reproduced in humans yet. Even though the two anti-herpetic drugs, valacyclovir, and acyclovir, were tested in a sufficient number of human participants, both yielded non-significant results, with only some favorable trends noted. Although viral infections are considered plausible factors involved in the etiology and pathogenesis of MS, the number and size of antiviral clinical trials in MS have been limited, and large, randomized controlled trials are needed to evaluate the role of antivirals in MS.

Although the use of antimicrobials in MS is still very limited, pwMS who are unwilling or unable to start on DMT, suffer from comorbidities that limit treatment options, or have exhausted DMT options might choose to initiate therapy with minocycline. The role of antimicrobials in progressive MS should be further explored, considering its pathophysiological differences from RRMS, the abundance of previous clinical trials that failed to produce encouraging results, and the modest effect on the progression of the available FDA-approved DMT [132,133,134,135]. Currently, there is only one FDA-approved DMT for PPMS and none for non-active secondary progressive MS [136]. Furthermore, immunosenescence may preclude the use of powerful DMT in the later stages of the disease, and subsequently, antimicrobials may offer an alternative solution [137,138,139,140]. As environmental factors, such as previous infections, could accelerate immunosenescence, antimicrobial agents might contribute to the deceleration of this process; therefore, they may lengthen the time interval during which pwMS can use DMT [141,142,143]. 

The role of microbiota in MS has recently attracted attention and has been the focus of MS research. Studies have highlighted that pwMS exhibit an imbalance in their gut microbial composition, referred to as dysbiosis, that is marked by a reduction in certain bacteria and an increase in others. This imbalance may promote inflammation and contribute to the autoimmune process underlying demyelination. Most of these gut bacteria are associated with specific metabolic pathways that contribute to the host’s immune equilibrium maintenance. Further research is needed to determine the role of gut microbiota and their metabolites in the susceptibility to and protection from the disease [142,143].

Animal studies strongly suggest that the gut microbiome has a substantial impact on the development of demyelinating diseases. Moreover, adjusting the composition of the microbiome has the potential to either worsen or alleviate symptoms. Various factors such as diet, insufficient vitamin D levels, smoking, and alcohol consumption have been linked to an increased risk of MS, and all these factors can influence the makeup of the gut microbiota. Antimicrobials affect the microbiota in different ways, and their effect has not been fully explored [144,145]. The effect of antimicrobials on microbiota can explain why antimicrobials may be an important area of study in MS. Understanding this intricate interplay between antimicrobials, microbiota, and MS could offer insights into potential therapeutic strategies, such as targeting the microbiome to modulate immune responses and potentially modify the disease course [146,147,148].

Despite some promising results, especially in the animal models of MS, the clinical efficacy of antimicrobials has not been fully established. To date, very few antibiotics and antivirals and only one antiparasitic agent have been tested in pwMS. Even though some antimicrobials, such as CEF and rifampycin, showed good results in animal models, they have never been tested in pwMS [149]. Given the small samples and short duration of the follow-up in most clinical trials, it is not yet possible to draw definite conclusions regarding the effect of antimicrobials on short and long-term outcomes. We hope this review article will serve as a stepping stone to facilitate the development of new clinical trials. Future research may expand previous observations and may include multicenter, well-designed, large-scale, randomized human trials testing but not limited to CEF, rapamycin, doxycycline, and HCQ. Implementing technology-assisted tools that have been shown to enhance clinical research and applying sensitive composite outcome measures can lead to successful clinical trials, especially in populations with progressive MS [150,151]. Digital innovation, as part of the clinical trials, can standardize outcome measures and reduce inconsistencies, thus assisting pwMS in monitoring their disease course and detecting new symptoms. Younger pwMS who are more familiar with digital technology can take full advantage of this approach [152]. Recruiting eligible participants might be somewhat challenging, especially for trials targeting a population with RRMS, given the numerous FDA-approved DMTs. In this context, adding antimicrobials to already approved DMT may solve this problem and increase recruitment. Currently, promising antiviral agents against EBV are under clinical trials and may open a new route for preventive and therapeutic interventions. Although vaccination is not the focus of this manuscript, vaccination might also be an effective preventive approach. Clinical research on antimicrobials is still in the very early stages, and further research is needed to provide insight into the potential benefit they can provide in pwMS. 

## 5. Conclusions

The number of published studies on antibiotics in MS is scant, and there are many limitations regarding their power and study design. Taking into consideration their shortcomings, we need to be extremely cautious in interpreting their findings. As we are awaiting the results of currently ongoing trials, we can conclude that the clinical trial results highlighted the role of minocycline as a potentially effective and safe antibiotic in MS management. HCQ success in PPMS could be a step forward in treating progressive MS. Rapamycin, beta-lactams, and antivirals yielded weak and inconclusive results. The quest is to identify antimicrobials that might optimize MS management and explore under which circumstances this goal can be achieved. Investigating the effect of antimicrobials on progressive MS could open a new line of research. The application of technology, effective collaboration between clinicians and basic science scientists, and well-designed studies are prerequisites to improve our understanding of the potential benefit of antimicrobials in MS.

## Figures and Tables

**Table 1:** Possible mechanisms of action and results from animal and human studies.

Drug	Possible Mode of Action in MS	Animal Studies	Human Studies
Beta-lactams	- Decrease glutamate neurotoxicity (GLT1 upregulation) - Modulate T-cell behavior - Reduce T-cell activation	- CEF reduces the severity of the EAE	- pwMS: CEF was not studied - PCN use (>2 weeks within 3 years before the first symptom) confers a 50% reduced MS risk - Higher MS risk with a 7-day course of PCN/antibiotics, suggesting infection as the causative factor
Minocycline	- Antiapoptotic - Anti-inflammatory - Anti-oxidative - Reduces T-cell infiltration into the CNS - Blocks MMP-2 expression - Inhibits microglial activation	- Reduces mean/cumulative EAE scores - Prevents dentate gyrus injury - No neurological symptoms in 50% of the minocycline-treated- EAE animals (day 14)	- Add-on to GA/interferon beta-1a: NS-but favorable trends - Monotherapy: significant decrease in ARR, GE lesions, BV loss, inflammatory cytokines - Significantly lower conversion of CIS to MS vs. placebo
Rapamycin	- Antiapoptotic - Promotes intracellular autophagy. - Inhibits the assembly of the subunits of the mTOR	Reduces clinical and histological signs of relapsing/ chronic EAE.	Reduces: - mean plaque area size - GE-enhancing lesions - NS decrease in EDSS (50% participants)
Antivirals	Suppresses the viral response in MS pathogenesis	Ganciclovir reduces microglia proliferation and severity of EAE.	- NS decrease in EDSS, new T2 formation - Subgroup with active baseline MRI (>1 GE) had fewer lesions/scan vs. placebo
Doxycycline	- Reduces peripheral inflammation	Not tested	- Add-on to INFb-1a: safe, well-tolerated; possibly effective in reducing ARR and disability. NS in MRI outcomes.
Hydroxychloroquine	- Inhibits microglial activation - decrease lipid peroxidation	Combined HCQ and minocycline (suboptimal doses): suppress symptomatology in chronic EAE	Improves T25FW in PPMS

PCN = penicillin, CEF = ceftriaxone, GA = glatiramer acetate, CIS = Clinically isolated syndrome, GLT1 = glutamate transporter 1, EAE= Experimental Autoimmune Encephalomyelitis, PwMS = people with MS, MMP= matrix metalloproteinases, mTOR= mammalian target of rapamycin, BV = brain volume, GE = gadolinium-enhancing, ARR = annualized relapse ratio, DMT = disease-modifying therapies, NS = non-significant, HCQ = hydroxychloroquine, T25FW = timed 25-foot walk.

**Table 2:** Risk of bias assessment. According to the Cochrane Handbook for Systematic Reviews of Interventions, version 5.1.0. +: low risk of bias; ×: high risk of bias; ?: unclear risk of bias.

	Random Sequence Generation (Selection Bias)	Allocation Concealment (Selection Bias)	Blinding of Participants and Personnel (Performance Bias)	Blinding of Outcome Assessment (Detection Bias)	Incomplete Outcome Data (Attrition Bias)	Selective Reporting (Reporting Bias)	Other Bias
Zabad et al., 2007 [13]	x	x	x	x	+	?	x
Alonso et al., 2006 [40]	?	?	x	x	x	?	?
Nørgaard et al., 2011 [41]	+	+	x	+	+	?	?
Sipilä et al., 2023 [43]	+	+	x	+	+	?	?
Metz et al., 2009 [70]	+	+	+	+	+	+	?
Sørensen et al., 2016 [71]	+	?	+	+	+	?	?
Metz et al., 2004 [72]	?	?	x	x	+	?	?
Zhang et al., 2008 [73]	x	x	x	x	+	?	x
Metz et al., 2017 [74]	+	+	+	+	+	+	+
Camara-Lemarroy et al., 2022 [75]	+	?	+	+	+	+	+
Minagar et al., 2008 [77]		x	x	x	+	+	x
Mazdeh et al., 2012 [78]	?	x	x	x	x	x	?
Bagherpour et al., 2018 [89]	x	x	x	x	?	?	?
Kappos et al., 2005 [90]	?	?	?	?	?	?	?
Gold et al., 2018 [108]	?	?	x	x	+	+	?
Lycke et al., 1996 [110]	+	?	+	+	+	+	+
Bech et al., 2002 [111]	+	?	+	+	+	+	+
Friedman et al., 2005 [112]	?	?	?	?	+	+	?
Koch et al., 2021 [115]	?	?	x	x	+	?	?
Sriram et al., 2005 [118]	+	?	+	+	+	+	+
Woessner et al., 2006 [119]	?	?	+	+	+	+	+

## Data Availability

Not applicable.
